# Aptamer-functionalized nanomaterials (AFNs) for therapeutic management of hepatocellular carcinoma

**DOI:** 10.1186/s12951-024-02486-5

**Published:** 2024-05-12

**Authors:** Xiujuan Yin, Jing Rong, Min Shao, Saisai Zhang, Likang Yin, Zhenqiang He, Xiao Wang

**Affiliations:** 1 Department of Radiology, The First Affiliated Hospital of Anhui Medical University, Hefei, 230022 Anhui China; 2 Clinical Medical College, Hebei University, Baoding, 071002, Hebei China

**Keywords:** Hepatocellular carcinoma, Aptamer, Aptamer-functionalized nanomaterials, Molecular imaging, Targeted therapy

## Abstract

Hepatocellular carcinoma (HCC) represents one of the deadliest cancers globally, making the search for more effective diagnostic and therapeutic approaches particularly crucial. Aptamer-functionalized nanomaterials (AFNs), an innovative nanotechnology, have paved new pathways for the targeted diagnosis and treatment of HCC. Initially, we outline the epidemiological background of HCC and the current therapeutic challenges. Subsequently, we explore in detail how AFNs enhance diagnostic and therapeutic efficiency and reduce side effects through the specific targeting of HCC cells and the optimization of drug delivery. Furthermore, we address the challenges faced by AFNs in clinical applications and future research directions, with a particular focus on enhancing their biocompatibility and assessing long-term effects. In summary, AFNs represent an avant-garde therapeutic approach, opening new avenues and possibilities for the diagnosis and treatment of HCC.

**Graphical Abstract:**

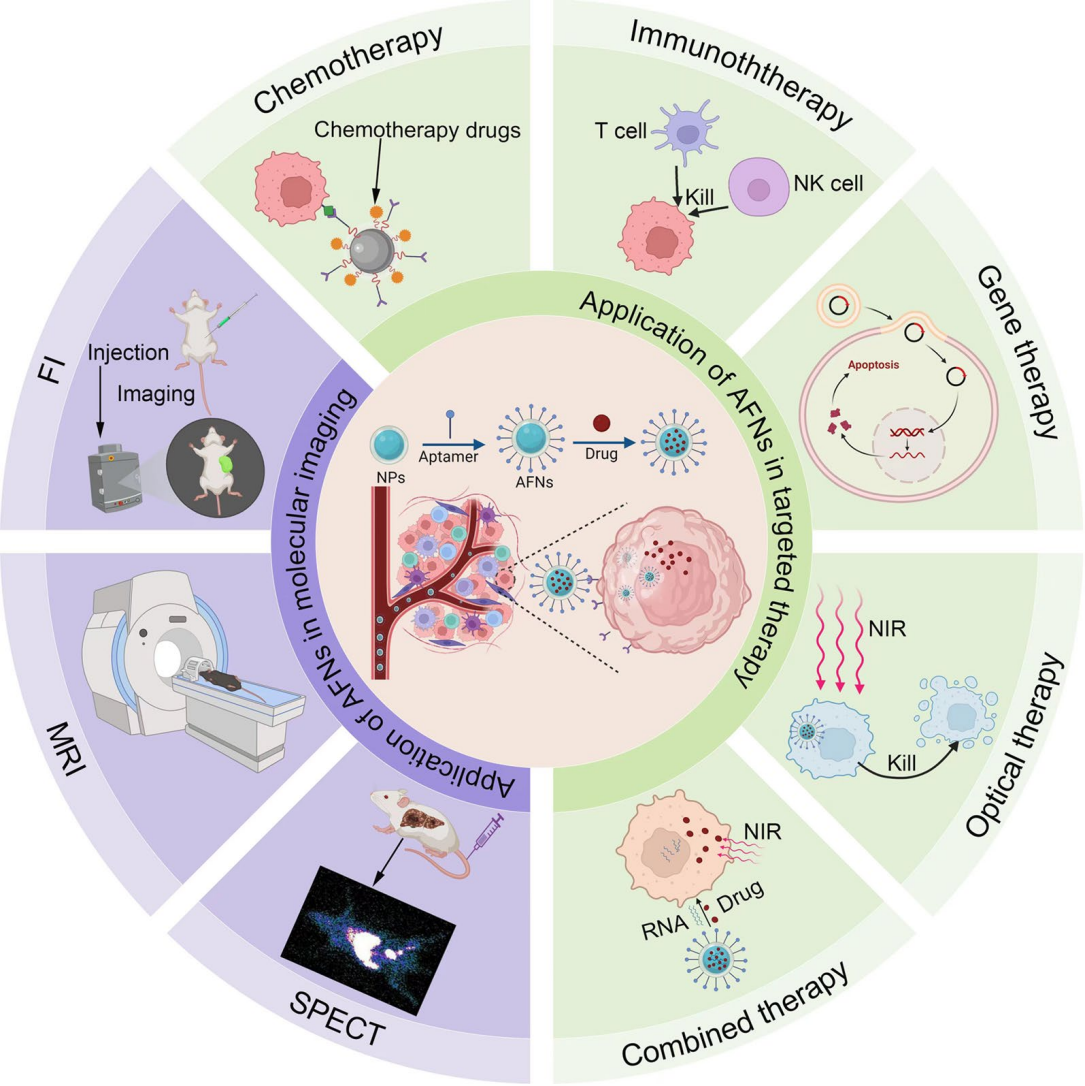

## Introduction

Hepatocellular carcinoma (HCC), one of the most prevalent malignant liver tumors worldwide [1], has seen significant advancements in recent years [2]. Although surgical resection and local ablation remain the primary treatment strategies for early-stage HCC patients, for those with intermediate to advanced stages, various systemic treatments have become crucial in improving prognosis. In recent years, targeted and immunotherapy have emerged as significant breakthroughs in the treatment of HCC, with Sorafenib (SRF) being the first approved targeted drug for advanced HCC, marking the inception of a new era in systemic therapy [3]. The introduction of new targeted drugs such as Lenvatinib [4], Ramucirumab [5], Cabozantinib [6], and immunotherapy agents like Pembrolizumab [7] and Atezolizumab [8] has further expanded treatment options. However, these emerging therapies, while effective for certain patient groups, present numerous challenges, including drug resistance issues [9] that limit the long-term efficacy of targeted drugs [10]. Secondly, while immunotherapy offers new hope for HCC treatment, it benefits only a subset of patients and can lead to unpredictable immune-related side effects [11]. Furthermore, the high cost of treatment and the complexity of therapeutic options pose challenges to patient decision-making. Hence, the search formore effective and personalized treatment regimens, along with addressing the challenges inherent in existing therapies, has become a focal point in current HCC research. This emphasis on innovation and the quest for tailored therapeutic strategies underscore the dynamic and evolving nature of HCC management, aiming to optimize patient outcomes while mitigating adverse effects and overcoming resistance mechanisms. In diagnosing HCC, traditional methods such as blood biomarkers and imaging techniques face limitations in both sensitivity and specificity, thereby hindering the accurate detection of early-stage HCC. For instance, alpha-fetoprotein (AFP), a widely used biomarker for HCC, does not consistently show elevated levels in the early stages of the disease [12]. Similarly, while imaging modalities like ultrasound (US), computed tomography (CT), and magnetic resonance imaging (MRI) prove somewhat effective in diagnosing later stages of HCC, they fall short in identifying smaller tumors and early lesions [13]. Consequently, there's a pressing demand for diagnostic approaches that offer enhanced targeting and specificity, enabling the early and accurate identification of small HCC lesions.

In contemporary cancer research and therapeutic arenas, aptamers emerge as cutting-edge technology, showcasing their distinctive value and potential for HCC treatment. These specialized single-stranded DNA or RNA molecules, or peptides, have garnered extensive study since the 1990s for their targeted recognition and binding to specific cancer cell surface molecules [14]. Produced through the systematic evolution of ligands by exponential enrichment (SELEX) technique [14, 15], aptamers bind to diverse targets, including small molecules, genes, proteins, and even whole cells or tissues [16, 17], enabling precise target identification. Aptamers offer numerous benefits over traditional protein antibodies, such as ease of synthesis, consistency, facile chemical modification, robust stability, and minimal immunogenicity or toxicity [18, 19], positioning them as superior biomedical tools. Particularly for HCC therapy, aptamers facilitate the development of targeted treatments that enhance drug efficacy and safety. Their diminutive size and molecular weight aid in navigating through and penetrating dense tumor matrices [20], crucial for optimizing drug delivery and minimizing adverse effects. Nonetheless, aptamers encounter significant hurdles in clinical settings, primarily related to their in vivo stability and bioavailability. Their small oligonucleotide nature makes them susceptible to nuclease degradation and rapid renal clearance, which has been a barrier to their broader clinical use [21, 22]. Recent advancements in material science, however, have opened novel pathways for leveraging aptamers in biomedical applications.

The swift progress of nanotechnology has ushered nanomaterials with distinctive properties into the realm of biomedicine [23, 24], notably garnering attention for their role in HCC management [25–27]. These materials, celebrated for their biocompatibility, biodegradability, stability under load, and advantageous surface area-to-volume ratio, offer novel avenues for boosting aptamer efficacy and presence in biological systems [28–30]. Modifying HCC-specific aptamers onto nanomaterials significantly prolongs their functional lifetime in vivo, thus broadening their clinical utility. AFNs, leveraging the precise targeting prowess of aptamers [31, 32], not only enhance target specificity but also shield aptamers from enzymatic degradation and premature renal clearance [33, 34]. This dual action facilitates deeper drug penetration, minimizes unintended cellular interactions, and amplifies treatment efficacy. Emerging as a powerful tool in HCC research, AFNs promise to revolutionize therapeutic delivery by directly targeting HCC cells with chemotherapeutics [35], gene therapies [36], or immunomodulators [37], thereby diminishing adverse effects on healthy tissues and optimizing therapeutic outcomes. Integrating AFNs with established treatment modalities enables tailored, more accurate interventions, particularly vital for combating drug-resistant HCC. In diagnostic applications, AFNs excel as potent imaging agents, enhancing MRI, CT, or single photon emission computed tomography (SPECT) modalities to reveal intricate details about tumor characteristics and microenvironmental conditions, crucial for accurate disease evaluation and tracking therapeutic progress. Through these multifaceted roles, AFNs embody an efficient, minimally toxic strategy, responding adeptly to the urgent call for refined HCC diagnostics and therapies tailored to individual patient needs.

Recent advancements have spotlighted AFNs in diagnosing and treating HCC, yet the research landscape shows a gap in their cohesive and integrative analysis. This review aims to consolidate aptamers tailored for HCC, exploring AFNs' attributes, clinical application barriers, and their recent breakthroughs in molecular imaging and targeted therapy. Initially, the review delineates the creation and optimization of HCC-targeted aptamers, emphasizing their role in elevating diagnostic and therapeutic precision through HCC cell specificity. Further, the discussion extends to AFNs' inherent advantages within HCC contexts. Moreover, an in-depth examination of AFNs' roles in diagnosis and treatment seeks to uncover existing challenges and anticipate AFN technology's trajectory. Through a comprehensive evaluation and discourse on AFNs' developmental and application strides, this review aspires to advance their contribution, offering HCC patients more accurate and efficacious diagnostic and therapeutic options.

## AFNs

AFNs have emerged as a breakthrough in HCC research, marking a new frontier in oncological biomedicine. By leveraging the targeted precision of aptamers and the versatile properties of nanomaterials, AFNs introduce an innovative approach to HCC therapy. Their capacity to selectively interact with specific molecular markers on HCC cells, including tumor-associated proteins and receptors, underscores their role in the meticulous identification and targeting of cancerous cells.

### Aptamer

AFNs have emerged as a powerful asset in HCC research, offering novel avenues for both diagnosis and therapy. Aptamers, with their precision in targeting biomarkers specific to HCC, such as cell surface proteins, enable targeted approaches to cancer treatment [38]. Their recent deployment in tumor identification [39], prognostic evaluation [40, 41], and therapeutic applications [42] underscores their utility in oncology. Among them, the aptamer A15, designed to target the CD133 + HCC cells, exemplifies the potential of aptamers for precise cancer cell targeting [43]. When integrated into nanomaterials, AFNs facilitate the delivery of therapeutic agents and function as diagnostic probes, enhancing the specificity and efficacy of HCC interventions [43]. This approach significantly improves chemotherapy outcomes by concentrating drugs in cancerous tissues, thereby minimizing adverse effects on healthy cells. For example, aptamer-modified micelles targeting the delivery of doxorubicin (DOX) and miR-519c have shown promise in synergistic HCC therapies, offering a balance between efficacy and safety [44, 45]. On the diagnostic front, aptamer-enhanced quantum dots [46] and gold nanoparticles [47] provide advanced solutions for early tumor detection and imaging, elevating diagnostic precision and sensitivity. This review methodically presents aptamers designed for HCC cell detection and their integration into therapeutic and diagnostic modalities (Table 1), paving the way for more targeted and efficacious applications of AFNs in the realm of HCC management.

**Table 1:** HCC targeting specific aptamer

Target	Aptamer	Chemistry	Sequences	Target cell line	Purpose	Refs.
GP73	GP73Apt	DNA	5′-GCAGTTGATCCTTTGGATACCCTGG-3′	–	Detection of GP73 in serum	[58]
GP73	A10-2	DNA	5′-ACGCTCGGATGCCACTACAG-TTGGTTTTTTTTTGTTATTTAGAGTAAAAACCTTGTGTGTAGTGA-CTCATGGACGTGCTGGTGAC-3′	–	Analysis of GP73 in human serum samples	[59]
Nucleolin	AS1411	DNA	5′-GTGGTGGTGGTTGTGGTGGTGGTGG-3′	HepG2	Therapeutic	[60]
GPC3	AP613-1	DNA	5′-TAACGCTGACCTTAGCTGCATGGCTTTACATGTTCC-3′	HepG2/Huh7	Diagnosis/therapeutic	[61]
GPC3	GPC3-3	DNA	5′CCTATTCCTTATTATATTTTCTTTTTTTGTAATTTGGTCG-3′	HepG2/Hep3B	Diagnosis	[53]
AFP	AFP	DNA	5′-GTGACGCTCCTAACGCTGACTCAGGTGCAGTTCTCGACTCGGTCTTGATGTGGGTCCTGTCCGTCCGAACCAATC-3′	–	Detection of APF in serum	[39]
Galectin-1	AP74	DNA	5′-TTACAGTCGGCTGTAATTTAGTGTATGTACCGGTGTGTGTACGAT-3′	HepG2	Therapeutic	[62]
Nuclei	TLS11a	DNA	3ʹ-AAAAAAACAGCATCCCCATGTGAACAATCGCATTGTGATTGTTACGGTTTCCGCCTCATGGACGTGCTG-5ʹ	H22	Diagnosis/therapeutic	[46]
TAG-72	TLS9a	DNA	5′-AGTCCATTTTATTCCTGAATATTTGTTAACCTCATGGAC-3′	HepG2/Huh-7	Therapeutic	[40, 63]
CD44E/s	CD44-Apt1	DNA	ATCCAGAGTGACGCAGCATCGCAACGATTAGTATGCACCCACCGTATAGGTTGGTCTCTGGACACGGTGGCTTAGT	Hep3B	Therapeutic	[64]
CKAP4	PS-ZL-7c	DNA	5′-C*G*C*A*G*CAAGGAGATTCGAGGGGGAAGGTTTGTTATAGGGGTTA*A*T*G*G*A-3′	HepG2/MDR	Diagnosis/therapeutic	[65]
EpCAM	EpCAM-Apt	DNA	5’-CACTACAGAGGTTGCGTCTGTCCCACGTTGTCATGGGGGGTTGGCCTG-3’	HepG2	Therapeutic	[57]
EpCAM	Ep166	DNA	5′-AACAGAGGGACAAACGGGGGAGATTTGACGTCGACGACAAAAAAAAAAAA AAA AAA AAA-3′	MHCC-LM3	Therapeutic	[66]
CD133	A15	RNA	5′-CCCUCCUACAUAGGG-3′	CD133 + BEL-7402	Therapeutic	[43]
Unknown	Apt-07S	DNA	5′-GTACTGTCAATTGGAAGTGGTGTTACGTTGTGTAGTCAAATCAGTGC-3′	HepG2/ SMMC-7721	Delivery of anticancer drugs	[67]
DKK1	D10	DNA	5′-TAGGGAAGAGAAGGACATATGATTAGGCCGTAAACGGGGCTAGGCGGGGATCATTGACTAGTACATGACCACTTGA-3′	–	Detection of Human DKK1	[68]

GPC3 is one of six mammalian members of the glypican family (GPC1-GPC6) [48, 49], highly expressed in HCC, but not in cholangiocarcinoma, gallbladder carcinoma, or benign liver tissue. Therefore, it may be a new serological marker for early detection of primary liver cancer, especially HCC [50, 51]. Aptaprobe is a functional and enhanced aptamer for target detection. As an oligomeric recognition molecule, it is used in a wide range of clinical sample diagnosis platforms, including blood, urine, and saliva. It is reported that aptamers bind to target molecules through the three-dimensional (3D) structure of sequences and their specific shapes [52]. Shin et al. synthesized and screened the aptamer GPC3-3 which can specifically bind to GPC3 target molecules, and constructed an aptaprobe platform based on a three-dimensional structure for detecting GPC3 [53]. The specificity of aptaprobe in the diagnosis of HCC was verified by Aptablotting and enzyme-linked immunosorbent assay (ELISA). In addition, the imaging based on aptaprobe shows that the binding characteristics and specificity of GPC3-3 remain unchanged in the xenograft model of liver cancer, which may provide a new idea for imaging diagnosis of liver cancer. The experimental results show that Aptaprobe has the potential to be used as an affinity reagent for detecting targets in vivo and in vitro diagnostic systems.

AS1411, arguably the most frequently used aptamer, has undergone substantive evaluation via phase II clinical trials for treating leukemia [54]. Recent reports are suggesting that AS1411 can specifically interact with nuclear proteins, which are influential biomarkers in numerous cancer cells and are significantly involved in the promotion of tumor growth and metastasis [55]. Therefore, the use of drug carriers that are modified with AS1411 has the potential to enhance drug delivery efficiency significantly, thus improving the therapeutic effects by targeting HCC cells proactively [56]. EpCAM, typically used as a tumor biomarker, is a transmembrane glycoprotein known for its self-renewing, highly tumorigenic, and genetic stability properties [57]. The high prevalence of EpCAM in HCC cells has brought about the potential for targeted HCC therapy through the construction of nanoparticles modified with EpCAM-targeting aptamers. Fast and efficient detection of alpha-fetoprotein (AFP) plays a crucial role in early liver cancer diagnosis. A study by Zhang et al. facilitated the grafting of an AFP-specific aptamer onto vertically-ordered mesoporous silica films, thereby constructing a cost-efficient and highly sensitive electrochemical aptamer sensor for the specific detection of AFP [39]. The validity and accuracy of this sensor were confirmed via the standard sample addition method, applying human serum.

### Characteristics and mechanism of AFNs

HCC stands as a predominant malignancy globally, challenged by hurdles such as elusive early detection, constrained therapeutic avenues, and prevalent relapse. The advent of AFNs in recent strides illuminates promising pathways for HCC's therapeutic and diagnostic realms. Marrying the precision targeting of aptamers with nanotechnology's versatile capabilities, AFNs carve out a distinct edge in advancing HCC management, showcasing a leap towards addressing the aforementioned treatment barriers.

AFNs herald a paradigm shift in HCC therapy, chiefly through their unparalleled targeting precision. Engineered to latch onto liver cancer cell-specific markers, such as GPC3 [61] and EpCAM [57], AFNs usher in tumor-focused therapy, sparing healthy cells and elevating treatment safety and efficacy. Furthermore, AFNs refine drug delivery, employing nanoparticles to shield and ferry therapeutics, thus ensuring stability and targeted release within the body. This mechanism significantly concentrates medications at the cancer site, exemplified by aptamer-equipped lipid nanoparticles that boost apigenin's tumor presence, mitigating systemic side effects and enhancing HCC patient outcomes [60]. Moreover, AFNs utilize tumor microenvironment-triggered release strategies, like pH or enzyme sensitivity [57], to unleash drugs precisely where needed, optimizing efficacy. Contrastingly, conventional chemotherapy's indiscriminate attack on proliferating cells, including benign ones, results in widespread adverse effects [69]. AFNs' targeted delivery and controlled release markedly diminish these detrimental impacts, offering a leap towards improving HCC therapy's tolerability and patient quality of life.

Beyond their role in directly administering therapeutics, AFNs also play a pivotal role in shaping the tumor microenvironment's immune landscape [37]. Specifically engineered to transport molecules that catalyze immune activation, such as immunostimulants [57] or immune checkpoint blockers, AFNs amplify the body's inherent tumor-fighting capabilities and potentiate immunotherapy outcomes. A groundbreaking approach involves a composite aptamer, integrating CTLA-4 and PD-L1 aptamers, serving as a dual-action immune checkpoint inhibitor. This innovative strategy obstructs both CTLA-4/B7 and PD-1/PD-L1 pathways, significantly bolstering the anti-tumor immune assault [57]. Moreover, these adept molecules facilitate a strategic congregation of T cells around the tumor locale, thereby intensifying their targeted attack on cancer cells, illustrating a sophisticated method to escalate immune-mediated tumor eradication.

AFNs extend their utility beyond mere drug conveyance, serving as invaluable assets in diagnostics and treatment tracking. By integrating aptamers with fluorescent markers, AFNs facilitate tumor tissue imaging [70], enabling precise evaluation of tumor metrics and responsiveness to therapies. In a notable application, aptamers were amalgamated with ultra-small superparamagnetic iron oxide (USPIOs), crafting AFNs with a knack for homing in on HCC cells. This combination enhances MRI's capability to divulge early-stage HCC details [71]. Moreover, an innovative diagnostic approach leveraging AFNs targets GP73, a hallmark molecule of HCC, for adept tumor cell imaging. This technique heralds a breakthrough in early HCC detection, enriching the diagnostic toolkit with a method that marries specificity with efficiency [58]. While AFNs have demonstrated significant promise for HCC treatment, they encounter critical hurdles, such as ensuring aptamer stability, boosting nanomaterial biocompatibility, and mitigating toxicity risks [72]. Addressing these challenges necessitates a concerted research effort aimed at innovating aptamer technology and refining nanomaterial fabrication. This direction entails both the discovery of novel aptamers and the advancement of nanomaterial design and synthesis processes, aiming to bolster AFNs' therapeutic effectiveness and safety profile.

Delving into the operational intricacies of AFNs in HCC therapy is pivotal for crafting targeted and efficacious interventions. AFNs stand out for their precision and effectiveness in both HCC management and early-stage detection, primarily through their targeted delivery and versatile functionalities. These attributes position AFNs as a potent solution to bypass the drawbacks inherent in conventional therapies. Additionally, their capacity to enhance early diagnostic precision promises to revolutionize tumor identification and intervention, potentially elevating patient survival outcomes significantly. As research deepens our understanding of AFNs' action mechanisms and their clinical implementations expand, AFNs are poised to be an indispensable asset in the evolving landscape of cancer treatment.

## Application of AFNs in molecular imaging

In the early stage of tumor formation, there might not be any discernible symptoms. Consequently, these tumors can progress to the mid to late stages before any symptoms manifest, thereby complicating treatment. For HCC, commonly used clinical diagnosis techniques include US, CT, MRI, positron emission computed tomography (PET), and SPECT. Ultrasound is the primary screening method due to its non-invasive nature, high safety, and low cost [73]. However, its depth limitation sometimes hinders the detection of tissue and organ lesions [74]. CT and MRI are regarded as the go-to diagnostic methods for HCC, employing contrast agents to enhance the examination and tumor detection. Despite their high sensitivity, PET/SPECT's specificity is typically low. To minimize patient harm from examination technology and increase examination accuracy, there has been burgeoning interest in molecular imaging technology.

**Fig. 1:**
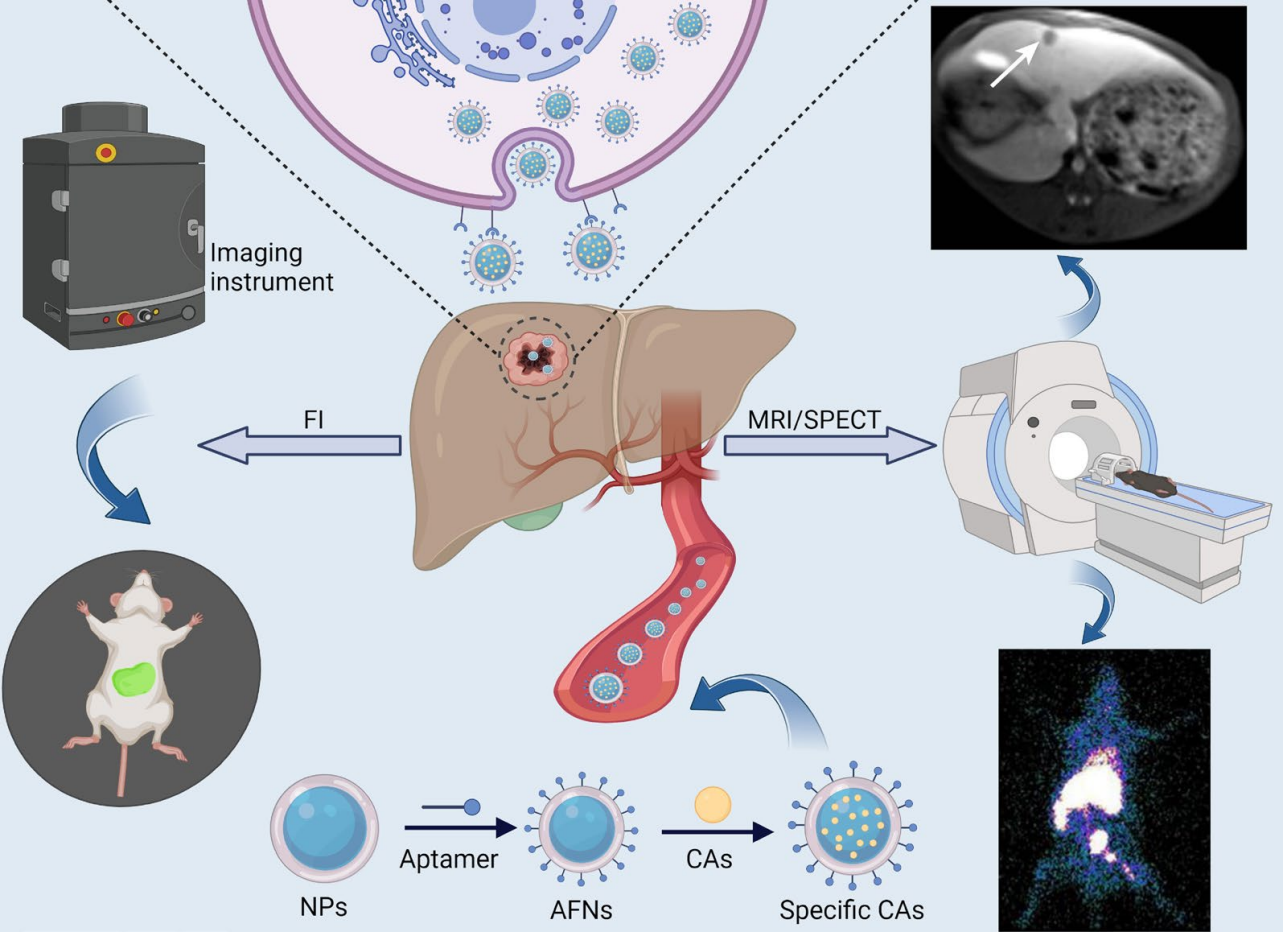
Schematic representation depicting the utilization of AFNs in molecular imaging of HCC. AFNs selectively target HCC cells by carrying imaging agents, leading to specific accumulation within tumor tissues. Subsequently, imaging is conducted using the corresponding imaging instruments, including FI, MRI, and SPECT

Molecular imaging was systematically introduced by Weissleder in 1999. It is a non-invasive visualization and quantitative description technology for genes, molecules, cells, tissues, and organs [75, 76]. It holds promising application prospects in early disease diagnosis. The imaging agent's ability to reach the tumor tissues or cells specifically and its non-toxicity to normal cells are vital for molecular imaging to diagnose accurately [22]. Considerable attention has been drawn towards the ultimate goal of optimizing aptamer-modified nano-materials loaded with imaging agents for HCC's targeted diagnosis. Aptamers serve as molecular imaging agents' target component as they can bind to a variety of macromolecules and nano-materials, asserting their potential clinical application [77, 78]. AFNs-mediated molecular imaging, which includes fluorescence imaging (FI), MRI, SPECT, and multimodal imaging, has garnered diverse exploration in HCC prevention (Fig. 1). Subsequently, we shall delve into the previous 5 years of experimental research in relation to AFNs in HCC molecular imaging focusing on FI, MRI, SPECT, and multimodal imaging (Table 2).

**Table 2:** Experimental study of AFNs in molecular imaging of HCC

Imaging	Aptamer	Nanocarrier	Labeling	Target cell line	In vivo/in vitro	Preclinical model	Refs.
FI	GP73	NGQDs	NGQDs	–	In vitro	–	[58]
FI	AS1411	AuNCs	AuNCs	HepG2	In vitro	–	[47]
FI	AS1411	CDs	Cy3/Cy5	HepG2	In vitro	–	[36]
FI	A15	SLNs	DiR	CD133 + BEL-7402	In vivo/in vitro	BALB/c + /nu mice model	[43]
FI	TLS11a	MSNs/Liposomes	DiR	H22	In vivo/in vitro	*S.* BALB/c mice model	[35]
FI	TLS11a	BMSF	Cy5	HepG2	In vivo/in vitro	BALB/c nude mice model	[106]
FI	EpCAM	HMSNs	Cy5.5	H22	In vivo/in vitro	*S.* Kunming mice model	[88]
FI	EpCAM	RNA/CD	Cy5	Huh7	In vivo/in vitro	*S./O.* BALB/c nude mice model	[89]
FI	AS1411	MSNs	FITC	HCCLM3/HepG2	In vivo/in vitro	*S.* C57BL/6 mice model	[70]
FI/MRI	TLS11a	GQDs/magnetic CS	ICG/Fe_3_O_4_	H22	In vivo/in vitro	*S.* BALB/c nude mice model	[46]
MRI	AP613-1	USPIOs	USPIOs	Huh7	In vivo/in vitro	*S.* BALB/c nude mice model	[71]
MRI	AP613-1	H-MnO_2_	Mn2 +	HepG2/Huh7	In vivo/in vitro	*S.* BALB/c nude mice model	[61]
SPECT	TLS9a	Polymeric NPs	^99m^Tc	HepG2/Huh-7	In vivo/in vitro	SD rats model	[105]

### FI

FI operates on the principle that photomultiplier tube detectors identify fluorescent groups which emit either visible light or near-infrared light. Typically, these fluorescent groups absorb a 650–1350 nm laser, a wavelength at which the laser penetrates tissue with greater depth [79]. With this range, near-infrared light delivers a resolution at the micron level in 0.2 mm-deep tissue [80, 81]. As a prevalent method in preclinical research, fluorescence imaging offers precise cellular and molecular-level information non-invasively. Notably, FI also allows for the evaluation of pharmacokinetic behaviors and biological distributions of targeted drugs or imaging agents, highlighting its broad application in nanomedicine, drug delivery systems, and diagnostic probe development.

Golgi Protein 73 (GP73), a serum biomarker for HCC, demonstrates superior diagnostic value relative to AFP. Existing detection methods for GP73, such as ELISA, chemiluminescence, electrochemistry, among others [82, 83]. Unfortunately suffer from limitations due to their stringent reaction conditions, low sensitivity, and high cost [84]. As a remedy, fluorescence analysis, a technique characterized by its high sensitivity, selectivity, and user-friendliness, has been adopted. Using fluorescence resonance energy transfer (FRET) technology, Liang et al., crafted a high accuracy GP73 fluorescence detection platform [58]. The platform deploys nitrogen-doped graphene quantum dots (NGQDs) paired with a GP73 aptamer (GP73Apt) as fluorescent probes and molybdenum disulfide@reduced graphene oxide (MoS2@RGO) nanosheets as fluorescence receptors. The MoS2@RGO nanosheets extinguish the fluorescence of NGQDs-GP73Apt through the FRET mechanism. Upon recognition of GP73 presence, NGQDs-GP73Apt binds to GP73, an action that creates a distancing effect, thus blocking the FRET process and restoring NGQDs-GP73Apt's fluorescence. Under optimal circumstances, the intensity of the detection system's fluorescence recovery aligns linearly with the GP73 concentrations within a 5–100 ng/mL range. Notably, the detection limit stands at 4.54 ng/mL (S/N = 3) and exhibits a high recovery rate (97.21–100.83%) in human serum samples. As a tool for early HCC diagnostic, the GP73 fluorescence sensing platform (dependent on NGQDsGP73Apt-MoS2@RGO FRET system) proves its effectiveness with significant accuracy, a broad output range, low expenses, and high specificity, thus possessing a promising clinical-application potential. However, a looming challenge resides in combating the high sensitivity of single fluorescence detection toward the environment, which is unfavorable for commercialization.

AS1411, a renowned DNA aptamer abundant in guanine, is known for its specific binding to certain tumor cells renowned for high nucleolin protein (NCL) expression [85]. The multifunctional NCL, a phosphorylated protein, is implicated in multiple cellular operations, encompassing transcription, packaging, and transportation of rRNA, along with DNA replication and recombination [86]. NCL has been pinpointed to have high expression levels in a bevy of malignant tumors, including gastrointestinal cell carcinoma, hepatocellular carcinoma, and human breast cancer [36, 85, 87]. Consequently, AS1411 can target HepG2 cells specifically for the FI of liver cancer. Zhao et al. utilized Cy3/Cy5 labeled carbon dots (CDs) as a carrier, amalgamating them with the AS1411 aptamer and siRNA for the targeted diagnosis and treatment of HCC [36]. The experimental studies indicate that CDs predominantly locate in the cytoplasm, emitting bright green fluorescence conducive for HCC diagnosis and successfully curtailing the migration and invasion tendencies of HCC cells. Additionally, Zhang et al. employed AS1411 aptamer as the targeting ligand of HCC, conforming to the fluorescent golden nanoclusters (AuNCs), and developed tumor targeting nanocarriers (AuNCs-CS-AS1411) with low molecular weight amphiphilic chitosan (CS) [47]. Subsequent to this, they encapsulated the anticancer drug methotrexate (MTX) into the nano-carrier, comparing the bifunctional nano-drug (MTX@AuNCs-CS-AS1411) with the human liver cancer cell line HepG2 and the normal liver cell line LO2 to demonstrate its comprehensive behavior. Their experiments divulged that MTX@AuNCs-CS-AS1411 boasts high fluorescence efficiency, photostability, low cytotoxicity, pH-dependent controlled release, and a remarkable sensitivity and targeting specificity for cancer cells. This establishes its viability for targeted FI diagnosis in tandem with cancer chemotherapy.

Chen et al. devised a graphene quantum dots (GQDs)/magnetic chitosan nanodelivery system (DOX-Fe_3_O_4_@CGA) further enhanced by the aptamer (TLS11a) [46]. This system, post-labeling with ICG, facilitates targeted drug deliveries and performs in vivo tumor FI, showcasing the real-time distribution of DOX-Fe_3_O_4_@CGA at the tumor site and assessing the treatment efficacy. Furthermore, the researchers undertook an in vitro MRI study, elaborately presented in the MRI section. Separately, Zhang et al. formulated a targeted gene-drug co-delivery system leveraging polyamidoamine-aptamer coated hollow mesoporous silica nanoparticles, followed by in vivo fluorescence imaging through the fluorescent dye Cy5.5 [88]. The co-delivery nano-system's tumor targeting capability became more enhanced post-aptamer integration, outperforming the accumulation resulting from the EPR effect. The fluorescence distribution in the tumor and prime organs validated the significantly enhanced tumor accumulation by co-delivery nanosystems. Ding et al.'s synthesized complex, TLS11a-LB@TATp-MSN/DOX, post-labeling with fluorescent dye DiR, enabled in vivo imaging in tumor mice, clearly showing the specific accumulation of TLS11a-LB@TATp-MSN/DOX in tumor sections [35]. Fluorescence imaging outcomes indicated that TLS11a-LB@TATp-MSN/DOX is a promising cancer tissue-specific and nuclear-targeted nano-drug delivery system. Chen et al. synthesized biological porous nanospheres (PRS) using RNA as carrier and cyclodextrin as a binder [89]. They deployed PRS with EpCAM-containing aptamer for targeted delivery. Upon Cy5-labeled PRS, widespread PRS aggregation in Huh7 cells and mouse tumor tissues was observable. Chen et al. constructed a novel "core–shell" co-assembled carrier-free nano-system based on natural ursolic acid (UA) and polyphenols (EGCG), useful for molecular imaging and HCC targeted therapy via the manipulation of EpCAM-aptamer and fluorescent dye Cy5 [57]. The experiments indicated significant fluorescence accumulation at the tumor site, confirming the synthesized nanoparticles' specific accumulation potential that enables real-time FI imaging and tumor-specific treatment, holding current applicability in targeted diagnosis and treatment of liver cancer.

### MRI

MRI, an in vivo imaging method borne from nuclear magnetic resonance, is heavily relied upon within the realm of liver cancer diagnosis. The high esteem in which it is held is primarily due to its international recognition as the standard modality for HCC diagnosis [90, 91]. MRI markedly outpaces other imaging methods, boasting superior safety levels, non-dependence on ionizing radiation, high spatial resolution, enhanced soft tissue contrast, and excellent penetration depth. These attributes render it a superlative tool in the accurate differentiation of HCC [92].

However, distinguishing early HCC nodules presents a quandary due to the limited specificity of MRI. This contributes to sub-optimal accuracy in the detection of nascent HCC [93, 94], particularly with small lesions, those less than 2 cm in size (HCC lesions < 2 cm) [95]. The implications are an increased likelihood of false negatives in distinguishing such nodules from benign ones [96]. The integration of molecular imaging with MRI to produce contrast agents with precise targeting capabilities could dramatically increase the accuracy of early HCC detection. Research has shown the use of AFNs loaded with contrast agents enables targeted MRI imaging. This approach has been noted in animal model studies employing AFNs-loaded Fe_3_O_4_ [97], superparamagnetic iron oxide nanoparticles (SPIONs) [98], USPIOs [71] and GdO [99]. Despite these being confined to cellular and animal level studies, they nonetheless offer hopeful insights and results.

Recent studies illustrate that the aptamer AP613-1 exhibits specific affinity for GPC3 positive HCC [100]. Zhao et al. constructed MRI probes that target HCC by synthesizing USPIOs with GPC3 specific aptamers [71]. The resultant Apt-USPIOs probe demonstrated stability and safety in tumor transplantation at the cellular level and exhibited GPC3 binding activity with T2 negative enhancement in vitro. The probe marks a promising trajectory for targeted MRI contrast agents. Furthermore, Wang et al., using aptamer AP613-1, developed a ligand that specifically targets HCC to create hollow mesoporous manganese dioxide (H-MnO_2_) nanoparticles modified by AP613-1 [61]. MnO_2_ nanosystems have been found to react with H^+^ and Glutathione (GSH), which are abundant in the TME [101]. The reaction results in the production of paramagnetic Mn^2^^+^ , significantly improving the contrast of T1 MRI. Additionally, the rapid excretion of MnO_2_ nanoparticles through kidney metabolism ensures there is no long-term in vivo toxicity. In vivo trials indicate the MnO_2_ nanosystem modified with AP613-1 exhibits high targeting performance and promising in vivo imaging results for ectopic tumor mice.

Chen et al. designed an aptamer (TLS11a)-modified graphene quantum dots (GQDs)/magnetic chitosan nanodelivery system (DOX-Fe_3_O_4_@CGA) [46]. This innovative system enables not only targeted drug delivery but also MRI imaging in vitro. When labelled with indocyanine green (ICG), DOX-Fe_3_O_4_@CGA facilitates in vivo FI, thereby rendering the real-time visualization of DOX-Fe_3_O_4_@CGA in the tumour site and allows for the evaluation of the therapeutic outcome. However, a key limitation of the study is its exclusive focus on the MRI imaging of tumour cells in vitro, neglecting an extensive examination of MRI imaging of tumour-bearing mice in vivo. Moving forward, the development of AFNs possessing superior imaging capabilities would furnish an effective resource for the early and accurate diagnosis of HCC.

### SPECT

SPECT is presently the most sophisticated molecular imaging method employed in clinical settings. The radionuclides utilized in this technology (^99m^Tc, ^123^I, or ^111^In) decay through single photon emission, which is directly detectable via a gamma camera. Contrasted against MRI, CT, or US, radionuclide imaging boasts greater sensitivity, albeit with lower specificity and spatial resolution [102]. Factors guiding the clinical selection of radiopharmaceuticals include minimal dosage, short half-life, and a lack of pronounced toxic and side effects on the body. These parameters also represent challenges that need to be addressed in the development of such drugs. Due to its high targeting proficiency, excellent tissue penetration, and rapid pharmacokinetics, the aptamer exhibits immense potential for application within radionuclide molecular imaging [103].

Dhara et al. designed non-visible nanoliposomes by conjugating them with tumor-sensing phosphorothioate and an amino-modified aptamer (AS1411). These nanoliposomes were then enveloped with apigenin to produce stable nanoliposomes (Apt-NLCs) [60]. The team conducted radiolabeling of the preparation using ^99m^Tc and administered the resultant ^99m^Tc-NLCS, ^99m^Tc-PEG-NLCS, and ^99m^Tc-APT-NLCS into a tumor mouse model. They subsequently observed the accumulation of these radiolabeled nanoliposomes using γ-scintigraphy 4 h and 8 h post-injection. Through different experimental procedures, the biological distribution of the nanoliposomes was assessed in various organs. The experimental data indicated that AS1411-functionalized NLCs led to significant accumulation of Apt-NLCs in tumor tissues. This can be attributed to the specificity of tumor hepatocytes towards AS1411, and resulted in notable drug retention in carcinogenic liver (Apt-NLCs > PEGNLCs/NLCs). TLS9a, a DNA aptamer of the mouse hepatoma cell line BNL 1ME A.7R.1, is a mere 39-nucleotide sequence that can specifically target hepatoma cells [104]. Chakraborty et al. developed a targeted phosphorylated ossification aptamer, TLS9a (L5). They modified a polymer loaded with paclitaxel using L5 (PTX-NPL5), then labeled the PTX-NPL5 system with ^99m^Tc for evaluation of the therapeutic impact of PTX-NPL5 [105]. Scintigraphy results showed that PTX-NPL5 significantly enhanced the therapeutic effect in chemotherapy-induced liver cancer in rats compared to the control group. This underlines the potential of AFN-mediated SPECT in the diagnosis of diseases, evaluation of therapeutic effects, and prognostication.

In molecular imaging, AFNs encounter distinct challenges across FI, MRI, and SPECT, each presenting unique avenues for advancement. FI's prowess in sensitivity is hampered by limited deep tissue visualization and autofluorescence interference. Future endeavors aim to craft next-generation NIR fluorescent probes and bolster AFNs' photostability and background discrimination. MRI's enhanced imaging specificity and contrast prompt the need to refine AFNs' magnetic properties for superior signal sensitivity, prioritizing biosafety and a viable route to clinical application. SPECT's challenge lies in stabilizing radioactive probes and minimizing dosage without compromising image quality. AFNs open pathways to safer isotope labeling, with prospective studies poised to elevate SPECT's targeting accuracy and resolution, concurrently diminishing patient radiation exposure.

To sum up, the potential for AFNs in molecular imaging is vast, yet realizing their clinical promise necessitates a collaborative, innovative effort across disciplines. The synergy of nanotechnology, biomedical engineering, radiology, and computer science is crucial for propelling AFNs advancements and their refinement. Future research agendas should prioritize evaluating the safety of AFNs systems and designing clinical trials that align with the nuanced requirements of clinical practice. By addressing these areas, the aim is to furnish tumor diagnostics and therapeutics, alongside other medical conditions, with superior imaging strategies, thereby significantly enhancing patient care and treatment outcomes.

## Application of AFNs in targeted therapy

HCC poses a significant challenge to global health, with the evolution of treatment strategies being a critical area of medical inquiry. The advent of AFNs marks a significant leap forward, particularly in targeted therapy, offering promising new avenues for HCC management. By merging nanotechnology's sophisticated drug delivery mechanisms with the precise targeting capabilities of aptamers, AFNs facilitate targeted attack on HCC cells while sparing healthy tissue. This dual functionality not only amplifies therapeutic outcomes but also mitigates adverse effects, heralding a revolutionary approach to HCC intervention. A notable instance of this innovation involves AFNs engineered to target GPC3, a key marker in HCC cells, demonstrating superior efficacy over traditional modalities [61]. Furthermore, AFNs' flexibility extends to gene therapy, as evidenced by their use in delivering the CRISPR/Cas9 system for precise genetic alterations within HCC cells [88], highlighting their potential to redefine cancer treatment paradigms. Despite the current preclinical phase of AFNs application in HCC, encompassing chemotherapy, immunotherapy, gene therapy, and photodynamic therapy (Table 3), the pathway to clinical translation beckons further exploration. This overview delves into AFNs' therapeutic roles, delineating the scope for clinical application and pointing towards a future where these nanomaterials could significantly impact HCC treatment strategy development.

**Table 3:** Experimental study of AFNs in targeted therapy of HCC

Therapy	Aptamer	Nanocarrier	Therapeutic Agents	Target cell line	Preclinical model	Summary	Refs.
Pharmaceutic treatment	AS1411	Liposomes	Apigenin	HepG2	SD rats model	Improvement cell apoptosis, reduction tumor incidences	[60]
	AP613-1	H-MnO_2_	SRF	Huh7/HepG2	BALB/c nude mice model	Inhibit the growth of tumors	[61]
	AS1411	AuNCs	MTX	HepG2	–	Increase the release of MTX in tumor cells	[47]
	TLS11a	MSN/Liposomes	DOX	H22	BALB/c mice model	Deliver DOX to the nuclei of tumor cells	[35]
	TLS9a	PLGA	PTX	HepG2/Huh-7	SD rats model	Reduce tumor incidences and tumor progress	[40]
	TLS9a	TPGS	PTX	HepG2/Huh-7	SD rats model	Inhibit the growth of tumors	[63]
	TLS9a	Polymeric NPs	PTX	HepG2/Huh-7	SD rats model	Superior therapeutic efficacy	[105]
	TLS9a	Phosphorothioate backbone	PTX	HepG2/ Huh-7	SD rats model	Inducing apoptosis, cell cycle arrest of tumor cells	[15]
	AS1411	PEG	VRM	HepG2/Huh-7		Extrinsic and intrinsic apoptosis tumor cells	[144]
Immunotherapy	TLS11a/CD16	Y-shaped DNA scaffold	TLS11a/CD16	HepG2	BALB/c nude mice model	Enhance adoptive immunotherapy of HCC	[114]
	CTLA-4 /PD-L1	DNA	P1/C4-bi-apt	Hepa1–6	BALB/c mice model	Inhibit tumor growth	[37]
	EpCAM-Apt	UA/EGCG	UA/EGCG	HepG2	KM mice model	Activate the innate immunity and acquired immunity	[57]
Gene therapy	AS1411	CDs	siRNA	HepG2	–	Enabling bioimaging and downregulation of gene expression	[36]
PDT	TLS11a	BMSF	BPQDs	HepG2	BALB/c nude mice model	Programmable killing of cancer cells in hypoxic TME	[106]
Combined therapy	TLS11a	GQDs/magnetic CS	DOX/GQDs	H22	BALB/c nude mice model	Inhibit tumor growth	[46]
	AS1411	Micelles	DOX/miR-519c	HepG2	BALB/c-nude mice model	Provide a strategy to obtain ideal anti-cancer efficacy	[44, 45]
	A15	SLNs	OXA/SAL	CD133 + BEL-7402	BALB/cnude mice model	Target carcinoma cells, evident antitumor effect	[43]
	EpCAM	HMSNs	CRISPR/Cas9/Sora	H22	Kunming mice model	Precise gene editing, synergistic inhibition of tumor growth	[88]
	EpCAM	RNA/CD	siRNA/SRF	Huh7	BALB/c nude mice model	Load various types of siRNA and drugs	[89]
	sLeX-AP	CNTs	Durvalumab/siRNA	HepG2	C57BL/6 mice model	Increase the apoptosis of HCC cells	[143]
	Ep166	Pharmacosomes	5′-DFUR/miR-122	MHCC-LM3	–	Enhanced the anti-proliferation, and modulated the cellular apoptosis	[66]
	AS1411	MSNs	ICT/FITC	HCCLM3/HepG2	C57BL/6 mice model	Detects nucleolin, and inhibits cell proliferation	[70]
	PD-L1/ EpCAM	USI	Sora/UA	HepG2/H22	H22 tumor bearing mice model	Realize the targeted delivery of dual drugs	[145]
	EpCAM	USFe^3+^ LA NPs	SRF/UA	HepG2	Unknown	Suppression of tumor growth and distant metastasis	[146]

### Chemotherapy

**Fig. 2:**
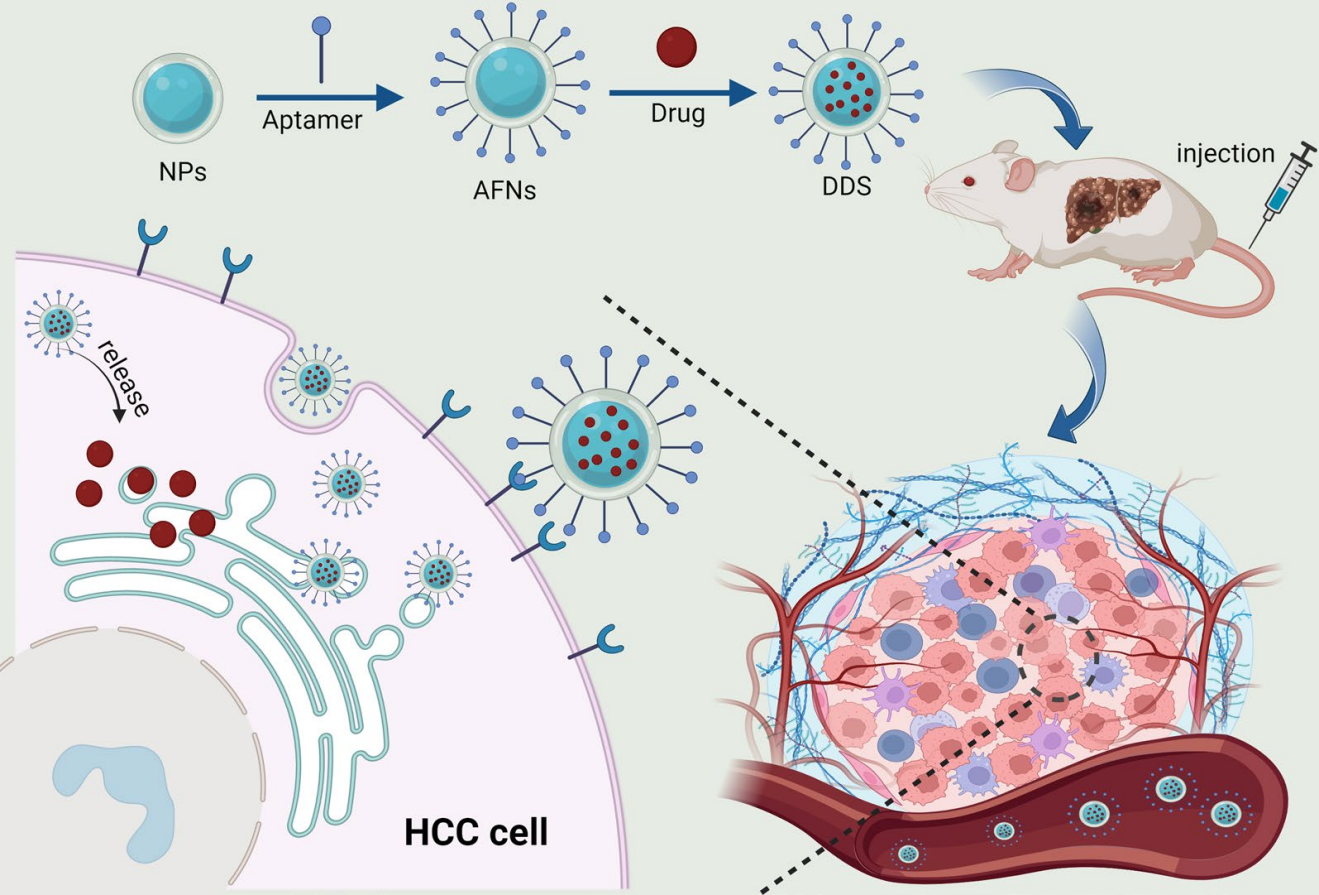
Schematic illustration of the mechanism by which AFNs facilitate targeted delivery of chemotherapeutic drugs to HCC tissues for treatment. AFNs, when loaded with chemotherapeutic agents, serve as specific drug delivery systems (DDS). By binding to specific receptors on the surface of tumor cells, AFNs achieve targeted delivery to the interior of tumor cells and release chemotherapy drugs for treatment

Chemotherapy is a cornerstone in the management of liver cancer, essential for systemic therapy [107]. Despite its role as a standard treatment for advanced stages, challenges such as drug resistance [108] and toxicity [69] persist, with few alternatives available. The evolution of AFNs introduces cutting-edge approaches to address resistance in HCC therapy. AFNs offer a novel method for administering both innovative [60] and established [47], chemotherapeutics, enhancing drug delivery and specificity to surmount resistance. These nanomaterials precisely direct drugs to tumor locales (Fig. 2), optimizing pharmacokinetics, and concentrating cytotoxic agents directly at the cancer site to mitigate systemic side effects [109]. A notable innovation involves the use of AP613-1 modified H-MnO_2_ nanoparticles for SRF encapsulation, resulting in the H-MnO_2_-SRF-APT system [61]. This system targets HCC cells with specificity, disintegrating in the tumor microenvironment to release SRF. Experimental outcomes in vivo showcased its potential in halting the growth of Huh7 xenografts in nude mice, affirming its biosafety and efficacy.

The investigation into the use of aptamer TLS9a featuring a thioskeleton alteration (L5) to selectively deliver treatment and induce apoptosis in tumor hepatocytes remains untouched. Chakraborty et al., hence, juxtaposed the therapeutic efficacy of drug nanocarriers enhanced with L5 (PTX-NPL5), with other experimentally-tested nanocarriers functionalized with both aptamers and non-aptamer ligands, such as galactosamine and transferrin [63]. The results demonstrated that the thioacid-modified L5 outperforms the traditionally-used experimental ligands for targeting liver cancer cells. PTX-NPL5, interestingly, exhibited no discernible toxicity to healthy hepatocytes, and therefore presents a novel and safer alternative for HCC targeted therapy. Additionally, the team compared the therapeutic effects of PTX-NPL5 and PTX nanoparticles (CF) combined with non-targeted reagent albumin on HCC [105]. A series of in vitro and in vivo experiments determined that PTX-NPL5 far outweighs CF in inducing apoptosis, inhibiting cell cycles, heightening the oxidative stress in tumor cells, and reducing liver cancer lesions. Contrarily to CF, PTX-NPL5 presented no apparent toxicity to healthy hepatocytes, further asserting the superiority of PTX-NPL5 over CF.

Apigenin, a plant-derived compound, has garnered attention in oncological research for its ability to induce cancer cell apoptosis, halt the cell cycle, reduce tumor invasiveness, and trigger autophagic and immune responses, underscoring its potential as an effective anticancer agent [110]. However, its clinical application is hampered by challenges such as poor water solubility, limited bioavailability, and nonspecific tumor targeting, which diminish its therapeutic utility [111]. Concerns over apigenin's adverse effects further complicate its use in cancer treatment. In response, AFNs have emerged as an innovative drug delivery approach. By employing nanoparticles functionalized with specific ligands, this strategy enhances drug delivery directly to the tumor site, ensuring targeted and efficient therapy, thus addressing the limitations of traditional apigenin administration. Dhara et al. developed pegylated nanoliposomes encapsulated by apigenin, functionalized by phosphorylated amino-modified AS1411 aptamers [60]. Their experiments, incorporating in vivo and in vitro models, investigated the drug internalization and apoptosis processes by analyzing the effects of bifunctional aptamer coupled PEG-nanoliposomes on PEGylated (non-aptamer coupled) or normal apigenin nanoliposomes. The study unraveled the in vitro processes facilitating the internalization of apigenin into hepatocellular carcinoma cells through the medium of modified AS1411 functionalized PEGylated nanoliposomes. Specifically, the internalization was mediated by nucleoprotein receptors, which ensured DNA damage in HCC to be irreversible. Moreover, the animal model experiments divulged a decrease in the clearance rate, an increase in drug accumulation in the tumor, and negligible toxic effect in the liver. These outcomes significantly bolstered cancer cell apoptosis, reaffirming the therapeutic potentiality of aptamer-coupled pegylated nanoliposomes in HCC treatment in comparison to unconjugated preparations. Consequently, this study has established the compelling advantages of leveraging modified AS1411 functionalized PEGylated nanoliposomes as an alternative drug delivery method to noticeably curtail the incidence of HCC tumors.

DOX, a primary agent in cancer chemotherapy, targets nuclear DNA. However, when used directly against liver cancer, its efficiency in nuclear transfer is limited. This efficiency, though, can be improved with targeted delivery. TATp, which binds specifically to the nuclear pore complex (NPC), facilitates the transport of macromolecules to the nucleus and allows the release of drugs carried by nanoparticles in the nucleus [112]. Ding et al. synthesised a TATp-mesoporous silica nanoparticles complex (TATp-MSN) and created liposomes (TLS11a-LB) carrying the liver cancer-specific aptamer, TLS11a. They then combined TLS11a-LB with DOX-loaded TATp-MSN to produce the compound TLS11a-LB@TATp-MSN/DOX [35]. This compound is an effective dual-target system capable of identifying both liver cancer tissues and nuclei of cancer cells in vivo. As a result of this targeting, DOX can be effectively released into the nuclei of liver cancer cells in vivo. Current research indicates that TLS11a-LB@TATp-MSN/DOX represents a significant step forward in cancer treatment, offering a promising tissue-specific, nuclear-targeted nano-drug delivery system.

### Immunotherapy

Immunotherapy, recognized as a leading-edge approach in HCC treatment [113], gains further momentum with the integration of AFNs, broadening therapeutic horizons. This cutting-edge strategy leverages immunotherapy's capability to modulate the immune system, significantly bolstered by AFNs' precision in targeting either the tumor cells or their surrounding microenvironment. AFNs stand out for their aptamer-mediated, highly specific targeting of HCC cellular markers and immune checkpoints like PDL-1 and CTLA-4 [37]. Such targeted delivery not only optimizes drug concentration at the tumor site for minimal non-target impact but also modulates immune checkpoint activity, invigorating the immune response against tumor cells. A pivotal advantage of merging AFNs with immunotherapy lies in AFNs' capacity to concurrently transport various immunomodulators to the tumor milieu [114], enhancing immunotherapeutic outcomes. This dual action—direct targeting by AFNs and immune system engagement—potentiates immunotherapy's effectiveness against HCC, mitigating associated side effects and heralding innovative treatment avenues.

UA, identified as a pentacycyclic triterpenoid compound, conveys the promise of anti-metastasis, anti-angiogenesis, anti-inflammation and anti-proliferation through thorough research on in vivo and in vitro cancer models [115, 116]. Moreover, compared with traditional chemotherapy drugs, UA is a natural product, that offers the benefits of low toxicity and high efficiency, garnering significant interest from the scientific community in recent years [117]. EGCG exhibits antioxidation and cardiovascular protective attributes, and it can also stifle the proliferation, migration, invasion, and metastasis of cancer cells [118]. Through extrapolating the natural origins of UA and EGCG, a novel "core–shell" co-assembled carrier-free nano-system was created by Zhang et al. via epcam aptamer modification for the synergistic treatment of HCC [57]**.** The nano-drug, acquired from edible plants, manifests excellent anticancer activity, low toxicity, impressive stability, a potent pH response and demonstrates a high penetration ability of tumor tissue. This uncomplicated and "green" method amplifies tumor cell uptake, extends circulation time, and essentially sidesteps the potential pitfalls synonymous with traditional carriers. The nanocomposite manifests low cytotoxicity within normal in vitro cells and displays superior biological safety and tumor accumulation efficiency in vivo. Additionally, the confluence of UA and EGCG can activate both innate and acquired immunity, imparting a substantial synergistic therapeutic effect. This study provides innovative concepts for future research and development of self-assembled delivery systems and offers effective therapeutic strategies for clinical HCC treatment.

T lymphocytes are critical in cancer immunotherapy, yet their anti-tumor immune response is often significantly impeded due to a paucity of tumor infiltrating lymphocytes (TILs) and considerable immunosuppression. Du et al. engineered a novel multifunctional aptamer, P1/C4-bi-apt, devised to bolster anti-tumor immunity [37]. This aptamer forms a highly stable circular structure and comprises a CTLA-4 aptamer and a PD-L1 aptamer, serving as an immune checkpoint inhibitor by concurrently blocking the CTLA-4/B7 and PD-1/PD-L1 signaling pathways to enhance the anti-tumor immune response. Additionally, it can bind to CTLA-4-expressing T cells and PD-L1-expressing tumor cells, thereby recruiting T cells to tumor sites and intensifying their immunocytotoxicity. Experimental findings demonstrate P1/C4-bi-apt's potential to substantially mitigate tumor growth and enhance the long-term survival rate of HCC-bearing mice. The research delineates a straightforward and efficient multifunctional aptamer that could be instrumental in the immunotherapy of liver cancer.

Adoptive cell immunotherapy utilizes immune effector cells to inhibit tumor growth or metastasis, thus emerging as a promising cancer treatment strategy [119]. However, problems like complicated in vitro culture and genetic engineering, potential life-threatening immune-mediated toxicity, and tumor heterogeneity, particularly in solid tumors, inhibit its widespread clinical implementation [120, 121]. A recent development shows bispecific aptamers (Ap) have the potential to modulate interactions between immune effector cells and tumor cells, marking them as promising therapeutic agents to specifically enhance cancer immunotherapy. Zheng et al. designed a stable, Y-shaped, bispecific Ap using a Y-shaped DNA scaffold to augment adoptive immunotherapy for HCC solid tumors [114]. This Y-type Ap, connecting HCC-specific Ap TLS11a with CD16-specific Ap through a Y-shaped DNA scaffold, is rigid, maintains considerable stability in 10% serum over 72 h, and resists the denaturing effect of 8 M urea. Moreover, it exhibits superior binding ability to NK cells and tumor cells both in vitro and in vivo, thereby triggering higher cytokine secretion and substantial anti-tumor efficiency. This study introduces a stable, bispecific Ap construction platform and holds significant potential in enhancing the adoptive immunotherapy of solid tumors.

### Gene therapy

**Fig. 3:**
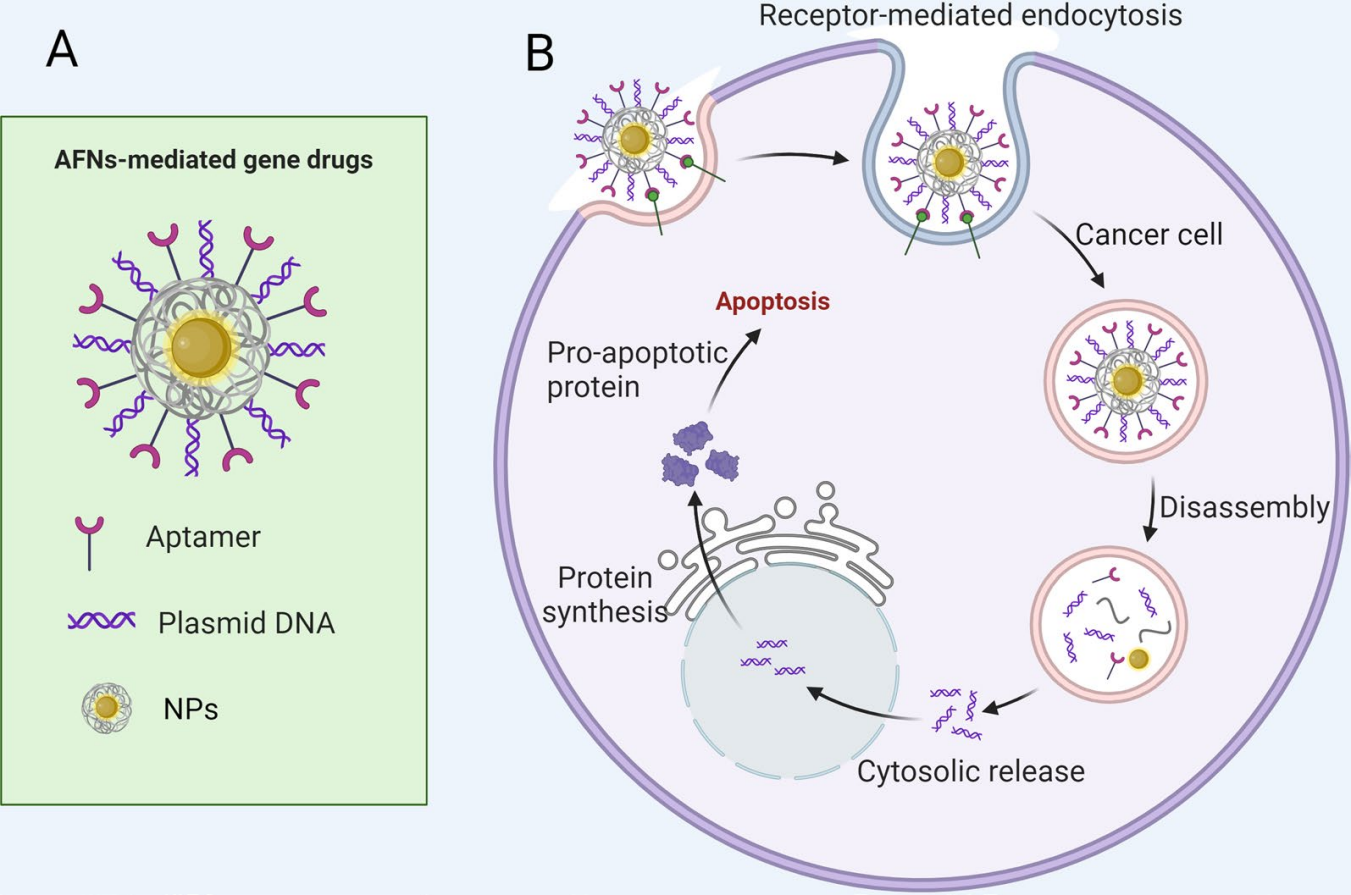
Shows the mechanism of AFNs in gene therapy of HCC. **A** Aptamer-modified nanoparticles loaded with gene drugs form an AFNs-mediated gene drug delivery system. **B** AFNs-mediated gene drugs bind to specific receptors on the surface of HCC cells and enter the cells, releasing gene drugs. This process induces apoptosis in cancer cells by regulating the expression of tumor genes

Gene therapy has recently been recognized as an innovative strategy for combating cancer and other persistent diseases [122]. The field has primarily focused on two gene vector categories: viral vectors and non-viral vectors, including cationic polymers and liposomes [123]. However, these vectors fall short of the ideal due to significant drawbacks such as eliciting undesirable immune reactions, exhibiting marked cytotoxicity, failing to shield nucleic acids from enzymatic degradation, and lacking precision in targeting [124]. Consequently, the quest for a gene delivery system that is safe, non-toxic, easily processed by the body, and capable of targeted gene delivery to disease sites is critical. AFNs stand out as a transformative solution in gene therapy, particularly for HCC. AFNs excel in accurately targeting tumor cells with gene vectors, facilitating the delivery of genes that can suppress or kill tumors or modulate the immune response directly at the affected sites (Fig. 3). This innovation not only elevates gene therapy's effectiveness but also substantially mitigates the cytotoxic and immunogenic concerns associated with conventional vectors [125]. A notable application of AFNs involves their use in targeting HCC cells with the CRISPR/Cas9 system for precise gene editing, targeting specific oncogenes [88]. This method underscores AFNs' role in advancing precise genetic interventions, heralding a new era of personalized cancer therapy.

The utilization of small interfering RNA (siRNA) for targeted gene silencing has emerged as a potential treatment for malignant tumors. However, the safe and effective delivery of siRNA to target cells remains a challenge. Accordingly, Zhao and colleagues proposed a solution, by synthesizing fluorescent CDs to serve as gene carriers in the siRNA delivery system, which then achieved an efficient gene knockout in vitro [36]. Further, upon combination with the aptamer AS1411, they were able to overcome the challenge of cell targeting. They discovered that CDs, which bear enough biocompatibility, can enhance cell uptake efficiency of siRNA. Their experimental findings indicated that the AS1411-mediated CDs/siRNA delivery system can effectively silence the expression of fragile X mental retardation protein, and successfully inhibit the migration and invasion tendencies of HCC cells. The novel approach of combining CDs with DNA/siRNA as a gene carrier not only offers an environmentally friendly synthesis method compared to traditional methods but also facilitates the preparation of complexes with high tumor cell selectivity and specificity. Consequently, these complexes warrant further exploration in cancer therapy, and their mechanisms of action need more comprehensive investigation.

The new gene editing technology, CRISPR/Cas9, shows improvements in targeting and gene silencing efficiency over siRNA, holding considerable promise for gene therapy [126, 127]. Nonetheless, significant challenges must be overcome before it can be clinically applied, including safety concerns and the potential off-target effects of the virus vector. In light of these obstacles, a non-viral tumor-targeting nano-delivery system could be promising for the safe application of the CRISPR/Cas9 system in gene-chemical combination therapies. Advances in nanotechnology have furnished robust delivery tools for the CRISPR/Cas9 system, enabling its rapid integration into oncology research [127]. Zhang et al. detailed a gene-drug co-delivery system (SEHPA) of hollow mesoporous silica nanoparticles (HMSN) coated with PAMAM-Apt, used to co-deliver sorafenib and CRISPR/Cas9 [88]. The core–shell nanoparticles displayed impressive stability and enabled ultra-high drug loading, targeted delivery, and controlled release of gene-drug combinations. SEHPA exhibited an EGFR editing efficiency exceeding 60% at all nine sites tested, with no off-target effects. Importantly, it was able to regulate the EGFR-PI3K-Akt pathway, inhibiting angiogenesis and synergistically suppressing cell proliferation. The system was successful in EGFR gene therapy, yielding 85% tumor inhibition in a mouse model. All the while, nanoparticles accumulated significantly in tumor sites in vivo, maintaining safety without harm to major organs. Given these properties, the nanoparticle system provides a general method for effective simultaneous loading of gene-drug combinations, enabling precise gene editing and synergistic tumor growth inhibition with minimal side effects on normal tissues.

The primary challenge of MDR becoming a treatment for HCC lies in the overexpression of the ATP-binding cassette (ABC) transporter family in cancer cells, including ABCG1, ABCC1, and ABCG2 [44, 45]. This overexpression leads to reduced sensitivity of cancer cells to chemotherapy drugs [128]. An effective way to reverse MDR and increase the sensitivity of cancer cells to chemotherapy drugs is to inhibit the expression of ABCG2, thereby blocking the drug efflux mediated by it [129]. Non-coding microRNAs (miRNAs) play a pivotal role in cellular differentiation, proliferation, and apoptosis by regulating mRNA expression [130]. The altered expression of miRNAs is a significant factor in acquiring MDR in HCC progression, with miR-519c showing strong correlation with MDR reversal by targeting ABCG2 mRNA [131]. Liang et al. fashioned AS1411 functionalized PEG-PLA micelle complex (MDPAS) for concurrent co-delivery of miR-519 and Adriamycin [44]. The MDPAS micelle has demonstrated superior tumor targeting ability by identifying the over-expressed nuclear protein of cancer cells and effectively internalizing the AS1411 aptamer-dependent pathway. Concurrently, the co-delivery of DOX and miR-519c through MDPAS micelles can reverse the MDR effect by inhibiting the efflux of ABCG2-dependent drugs and increasing the intracellular concentration of DOX as a result enhancing the inhibition of tumor growth and achieving optimal therapeutic effect. Consequently, MDPAS micelles hold promise for the amalgamation of chemotherapy and gene therapy and present a potential method for the treatment of cancer, primarily heavily drug-resistant tumors.

### Optical therapy

**Fig. 4:**
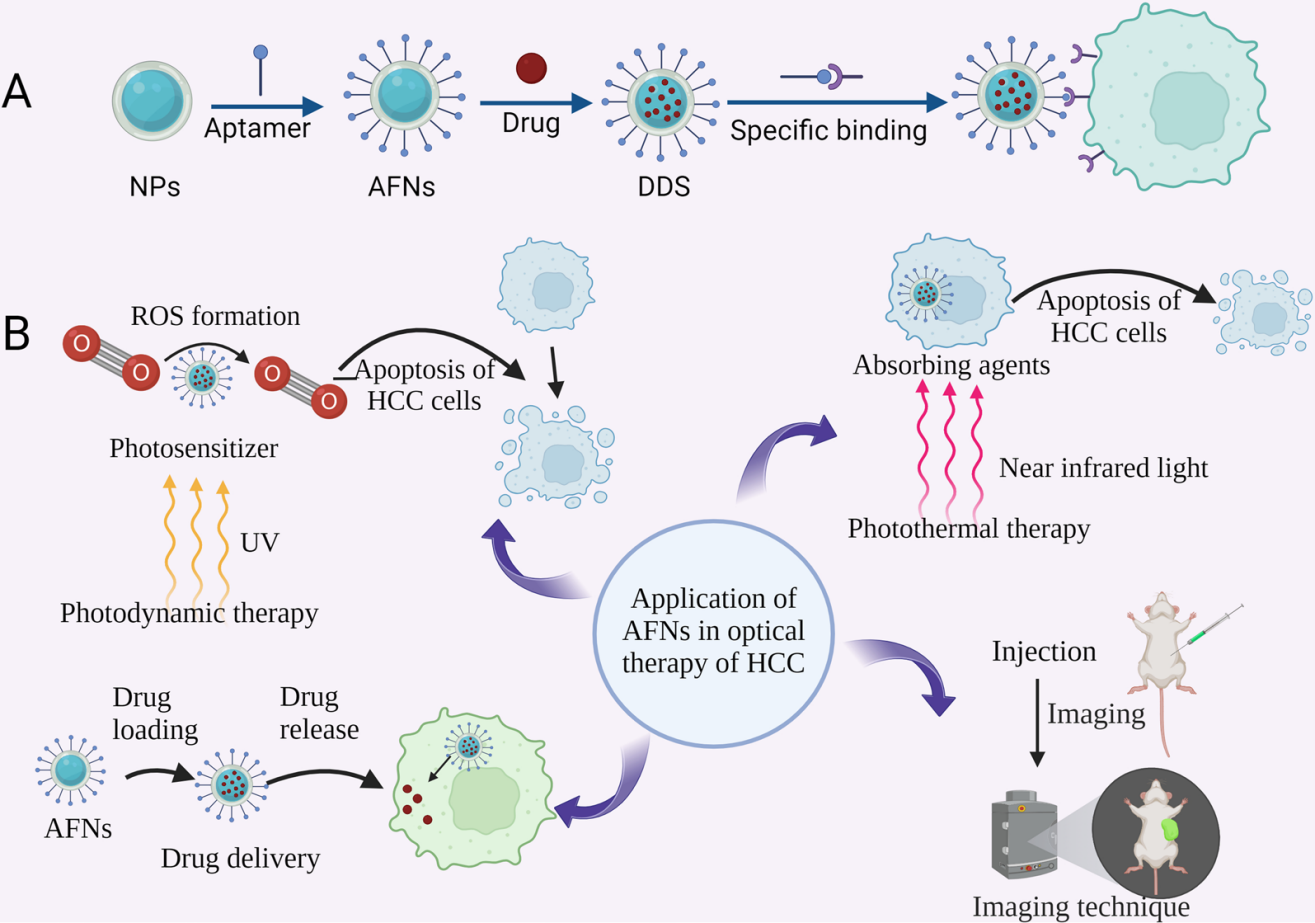
Demonstrate the application and mechanism of AFNs in HCC in optics. **A** Schematic diagram illustrating the synthesis process of AFN-mediated optical drug delivery system. **B** Illustration of AFNs application in HCC. AFNs, as drug delivery systems, can specifically deliver photosensitizers into HCC cells, which, upon laser irradiation, generate ROS or heat to induce HCC cell death. Additionally, the photosensitizer emits fluorescence for fluorescence imaging

Phototherapy, encompassing photodynamic (PDT) [132] and photothermal therapies (PTT) [133], emerges as an innovative approach to HCC management. The efficacy of such treatments hinges on the precision targeting of photosensitizers to tumors, meticulous control over drug release to maximize therapeutic outcomes, and minimization of adverse effects. Within this context, AFNs have demonstrated significant potential for HCC phototherapy (Fig. 4). Their primary advantage rests in the utilization of aptamers' specificity for direct HCC cell targeting, thereby facilitating focused delivery of phototherapeutic agents to tumor sites. This not only ensures elevated agent concentrations within tumors but also mitigates damage to adjacent healthy tissues. Specifically, AFNs serve as efficient carriers for photosensitizers to tumors, exemplifying an optimal platform for PTT. Chen et al.'s development of an aptamer-modified graphene quantum dots (GQDs)/magnetic chitosan nanosystem for combined PTT and chemotherapy [46] illustrates this capability. This innovative system targets HCC cells to deliver chemotherapeutic drugs, like DOX, and photosensitizers, such as GQDs, directly to neoplastic sites. Utilizing GQDs' photoreactivity, the DDS transforms light into heat upon irradiation, effectuating PTT. Beyond enhancing PTT efficacy, AFNs also thwart chemotherapy resistance by meticulously modulating drug release. The incorporation of magnetic chitosan into DDS augments its drug encapsulation, acid-sensitivity, and tumor-imaging properties, rendering the therapy both controllable and observable. This AFNs-centric approach to phototherapy signifies a sophisticated method for HCC's precise management, with empirical evidence underscoring its capacity to notably suppress tumor progression and extend survival in tumor-bearing models, thereby affirming AFNs' substantial promise for synergistic cancer therapeutics.

Recent research highlights the usage of BPQDs within the biomedical domain, largely owing to their superior photocatalytic activity in PDT and comprehensive light absorption, spanning from ultraviolet (UV) to NIR in PTT [134, 135]. Nonetheless, the environmental instability linked to the hypoxic TME has significantly interrupted the biological application of BPQDs, primarily in oxygen-dependent PDT. Lan et al. constructed a BPQDs hybrid nanocatalyst enhanced by HCC-specific targeting aptamer TLS11a; this could target liver cancer cells and self-supplement oxygen (O_2_) to hypoxic TME, thereby augmenting PDT efficiency [106]. Subsequently, the researchers prepared the BPQDs hybrid mesoporous silica framework (BMSF) and in-situ synthesized platinum nanoparticles. In contrast to PEG-BMSF@Pt without H_2_O_2_ incubation, the nano-system (Apt-BMSF@Pt) enhanced by TLS11a aptamer/Mal-PEG-NHS simulated a closed system of the PEG-BMSF@Pt nano-catalyst when H_2_O_2_ and a near-infrared laser were present. In a mouse model, Apt-BMSF@Pt effectively accumulated at the tumor site; the BMSF core functioned as a photosensitizer, generating reactive oxygen species while the PtNPs acted as a catalyst converting H_2_O_2_ into O_2_. Consequently, this self-compensated the hypoxia TME and boosted the PDT. The examination of tumor volume/weight, Hematoxylin & Eosin (H&E), and immunohistochemical analysis revealed that Apt-BMSF@Pt had significant antitumor effects and minimal side effects in vivo**.** Apt-BMSF@Pt can act as an active targeting nano-catalyst demonstrating exceptional environmental stability, specific targeting ability for HCC cells, and a self-compensation feature of oxygen. Thus, it holds potential for self-regulation of precise cancer phototherapy within a hypoxic TME. For the code, please specify instructions and details like the programming language, the task, and code issue.

### Combined therapy

**Fig. 5:**
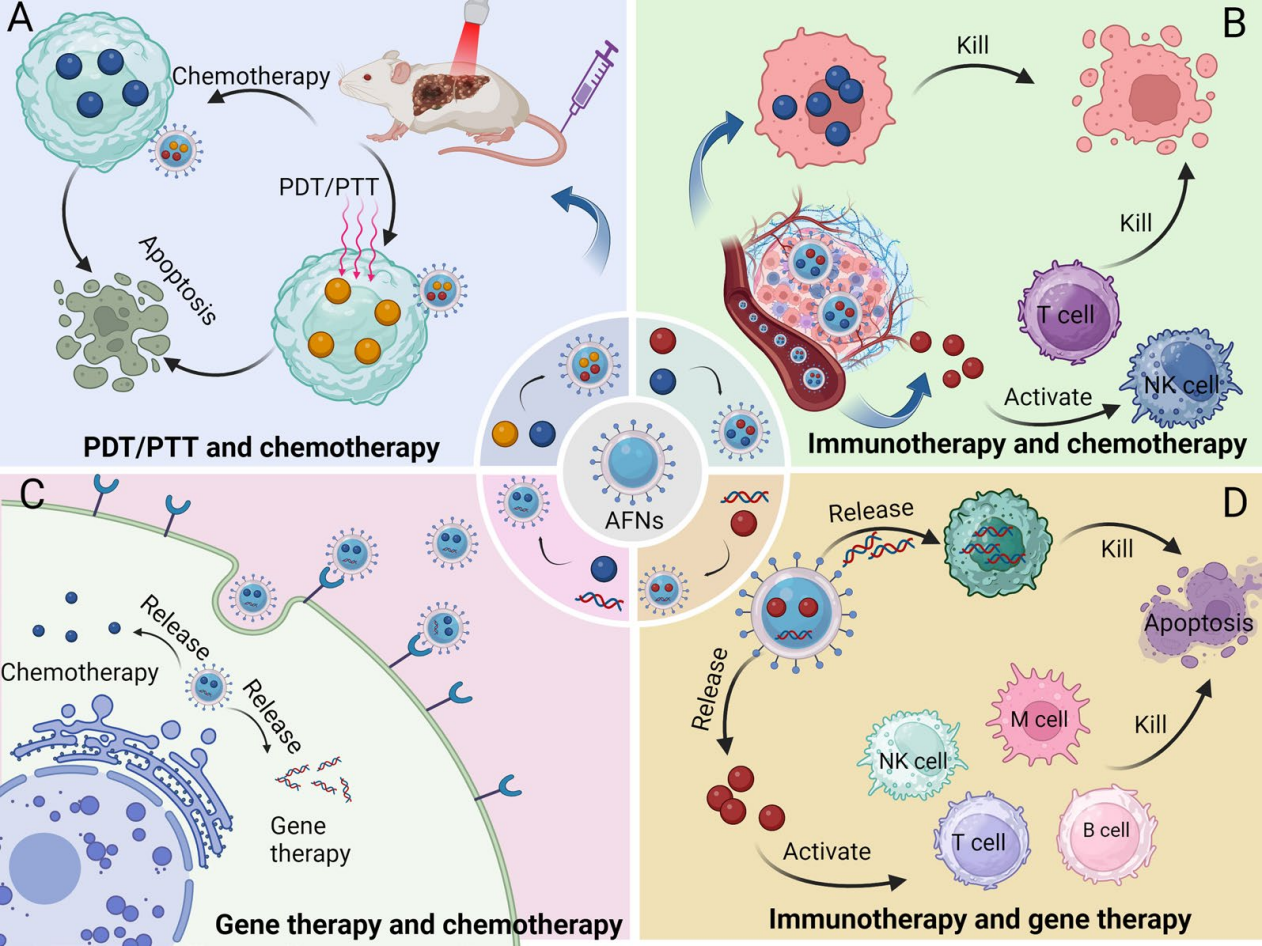
Schematic illustration of application of combined therapy in HCC. **A** Schematic diagram illustrating the co-delivery of photosensitizers and chemotherapeutic drugs by AFNs for combination therapy. **B** Schematic diagram illustrating combination therapy of immunotherapy and chemotherapy drugs. **C** Schematic diagram illustrating combination therapy of gene therapy and chemotherapy drugs. **D** Schematic diagram illustrating combination therapy of immunotherapy and gene therapy drugs

Combination therapy has emerged as a pivotal strategy against drug resistance and for amplifying the efficacy of treatments across various medical conditions. Within the realm of HCC management, the advent of AFNs has unveiled innovative avenues to transcend the constraints inherent to monotherapies, thereby enhancing therapeutic outcomes. AFNs, leveraging the precision targeting of aptamers alongside nanotechnology's versatile functionalities, herald a novel paradigm in HCC therapeutics, particularly emphasizing synergistic interventions and precision in treatment delivery. Distinctively, AFNs excel in accurately conveying a spectrum of therapeutic modalities—ranging from conventional chemotherapeutics and cutting-edge immunotherapeutic agents to photosensitizers and gene-based treatments—directly to HCC cells or their microenvironment (Fig. 5). This capability substantially raises the local concentration of therapeutics at the tumor site, intensifying the assault on cancerous cells while sparing healthy tissues, thus mitigating adverse effects. Additionally, AFNs' role in composite therapeutic schemes underscores their potential to amplify the benefits of chemotherapy, immunotherapy, phototherapy, and gene therapy through cooperative mechanisms. An illustrative example is the dual functionality of aptamers for both targeting the delivery of chemotherapeutic agents like Sorafenib (SRF) and facilitating gene editing via siRNA, thereby orchestrating a multifaceted attack against HCC [89], exemplifying the comprehensive therapeutic prowess of AFNs.

Solid lipid nanoparticles (SLNs) are solely composed of solid lipids, encapsulating lipophilic drugs in a specialized lipid matrix to control their release. These nanoparticles are emerging as an efficient lipophilic drug carrier, known to enhance drug bioavailability, ensure drug protection, and prolong drug release time, with the added benefits of non-toxicity and low cost [136]. Lv et al. employed a sequential therapeutic strategy involving A54 peptide-modified SLNs carrying Oxaliplatin (OXA) targeting extratumoral cells, and A15 aptamer-modified SLNs loaded with salinomycin (SAL) aiming at CD133^+^ tumor stem cells [43]. They developed two modular lipid delivery systems (A54-PEG-SLN/OXA and A15-PEG-SLN/SAL) for specific hepatocellular carcinoma management. In-vitro and in-vivo results indicated that A54-PEG-SLN/OXA targets BEL-7402 hepatocellular carcinoma cells with high precision and exhibits strong anti-tumour efficiency. A15-PEG-SLN/SAL targets and penetrates spheres composed of CD133^+^ cancer cells. Experimental findings confirmed that A54-PEG-SLN/OXA effectively eradicates tumour cells, exposing CD133^+^ cancer cells. Upon administering A15-PEG-SLN/SAL, tumour growth was significantly inhibited. The dual use of A54-PEG-SLN/OXA and A15-PEG-SLN/SAL offers a tailored approach, enabling sequential and timely application with remarkable anti-tumour outcomes.

MDR poses a significant challenge to cancer treatment. However, combining gene therapy with chemotherapy could potentially amplify the effects of cancer treatment by reversing MDR. This combination can enhance the sensitivity of cells to chemotherapy drugs and effectively combat MDR. For instance, Liang and colleagues devised an MDPAS micelle capable of reversing MDR. This is achieved by inhibiting the efflux of ABCG2-dependent drugs and elevating intracellular DOX concentration via miR-519c, subsequently inhibiting tumor growth and achieving desirable therapeutic effects in HCC animal studies [44]. Moreover, Zhang et al. designed a SEHPA system tailored to target EGFR proteins, which concurrently delivers SRF and CRISPR/Cas9. This results in efficient EGFR gene therapy and suggests a standard delivery method for effective gene-drug combination loading [88]. Furthermore, Chen and colleagues synthesized PRS using RNA as the carrier and cyclodextrin as the adhesive [89]. PRS incorporates aptamers for targeted delivery and siRNA for EpCAM gene silencing, in addition to cyclodextrin's capacity to load insoluble SRF through its hydrophobic cavity. With aptamers' assistance, porous nanospheres can penetrate targeted HCC cells before being degraded by cytoplasmic-Dicer enzymes. This process releases siRNA and sorafenib for simultaneous treatment, and the synergistic effect of PRS has been confirmed through in vitro functions, subcutaneous tumor-bearing mice, and in situ tumor-bearing mice in vivo models. Owing to the promising perspective of the synergistic effects of gene therapy and chemotherapy, along with the broadening use of RNA and cyclodextrin of porous nanospheres to carry a variety of siRNA types and small-molecule drugs, the biological porous nanospheres offer an opportunity for targeted drug delivery specifically tailored for treating certain types of tumors.

The prodrug 5ʹ-deoxy-5-fluoropyridine (5'-DFUR) is converted into 5-fluorouracil (5-FU) under the influence of pyrimidine nucleoside phosphorylase (PYPASE) [137], which has a degree of tumor specificity. However, it should be noted that 5-FU, derived from 5'-DFUR, exhibits lackluster specificity for tumor tissues, and is associated with adverse reactions including liver dysfunction and metabolic disorders [138]. Moreover, HCC typically shows resistance to conventional chemotherapy. MicroRNA-122 (miR-122), abundantly expressed in the normal liver, is typically utilized in conjunction with docetaxel, exhibiting tumoricidal susceptibility and anti-proliferative activities [139]. To further this line of research, Xue et al. developed a novel drug delivery particle using 5ʹ-DFUR, coupling it with plasmid DNA encoding miR-122 and anti-EpCAM aptamer via hydrogen bonds to form a nanocomposite p122-ap1@NPC-D [66]. This nanocomposite successfully transfected miR-122 into MHCC-LM3 cells through the controlled release of TMEs (marked by high levels of H_2_O_2_ and low pH), subsequently releasing 5-FU. Furthermore, p122-ap1@NPC-D notably countered chemotherapy resistance and exhibited a synergistic effect. These nanoparticles have the potential to significantly amplify anti-proliferative abilities at the cellular level and modulate cell apoptosis via downregulation of various signaling pathways, indicating promising applications for HCC treatment. Nevertheless, these findings are primarily based on in vitro studies, further in vivo investigations are required for the verification of the targeting and synergistic therapeutic impact of p122-ap1@NPC-D.

CNTs possess exceptional tissue permeability, which allows them to penetrate numerous biological barriers [140]. Moreover, their unique nanostructure endows them with substantial specific surface area, enhancing their capacity for high drug loading [141]. Research has revealed that CNTs can penetrate tumor necrosis and tuberculous ulcer tissue, thereby ensuring targeted delivery of chemotherapy drugs, yielding substantial therapeutic effects [142]. Niu et al. developed Durvalumab/CNT/PEI/aptamer-siRNA chimera (chimera/Durmab/CNT) nanoparticles for synergistic therapy of HCC [143]. In vitro experiment revealed that the aptamer-siRNA chimera can specifically bind to HCC cells, suppress the expression of the trigger receptor-2 (Trem2) expressed on myeloid cells, elevate the T cells to CD8^+^ T cells ratio, leading to enhanced apoptosis of HepG2 cells. Transmission electron microscope results confirmed that the CNT/PEI nanoparticles can regulate the prolonged release of Durvalumab for up to 48 h. Moreover, in vivo experimental outcomes revealed that the chimera/Durmab/CNT could significantly curb the proliferation of transplanted tumors in murine models. These studies affirm the efficacy of Durvalumab/CNT/PEI/chimera in treating HCC by stimulating anti-tumor immunity, offering a new approach to liver cancer immunotherapy.

The most prevalent methods for AFNs-mediated combination treatment for HCC include drug synergistic therapy, gene-chemotherapy synergistic therapy, and chemotherapy-immunotherapy. Chen and colleagues crafted a novel therapeutic approach by integrating chemotherapy with PTT for enhanced HCC treatment. They developed a TLS11a-functionalized GQDs/magnetic chitosan nanosystem capable of delivering the chemotherapy drug DOX and photosensitizer GQDs to tumor tissues. Serving as a photosensitizer, GQDs can effectively manage PTT and prevent DOX release into the bloodstream. Studies have demonstrated that magnetic chitosan nanosystem significantly restricts tumor growth and extends the lifespan of tumor-bearing mice, indicating its robust potential for synergistic tumor therapy.

In a related development, Xiang and colleagues invented a unique nucleoprotein-responsive nanoparticle platform for liver cancer's cooperative treatment. The researchers amalgamated the AS1411 aptamer, icartin (ICT), and fluoresceine isothiocyanate (FITC) with mesoporous silica nanoparticles, creating Atp-MSN (ICT@FITC) NPs [70]. The targeted nucleoprotein's binding to AS1411 induced separation from the surface of mesoporous silica nanoparticles, instigating the release of FITC and ICT. This enables detection of nucleolin by monitoring fluorescent intensity. Impressively, Atp-MSN (ICT@FITC) NPs not only curbed cellular proliferation but also augmented the ROS level to stimulate the Bax/Bcl-2/caspase-3 signaling pathway, resulting in cell apoptosis both in vitro and in vivo. Of note, the researchers identified lower toxicity levels with Atp-MSN (ICT@FITC) NPs, which also triggered infiltration of CD3^+^ T cells. Consequently, Atp-MSN (ICT@FITC) NPs may offer a reliable and safe platform for the diagnosis and concurrent treatment of liver cancer.

## Conclusion

Ongoing scientific exploration has unveiled the substantial application potential of AFNs in treating HCC. This innovative approach capitalizes on the versatility of nanomaterials combined with aptamers' precise targeting capabilities, introducing a groundbreaking strategy for combating HCC. Specifically, the integration of particular aptamers with various nanoparticles, such as liposomes [60], CDs [36], and micelles [44, 45], facilitates targeted recognition and efficient drug delivery to HCC cells. This method significantly elevates drug concentration in tumor cells, enhancing therapeutic effectiveness while markedly diminishing adverse effects on normal liver cells. This application exemplifies the transformative potential of accurately guided drug delivery systems in revolutionizing HCC treatment modalities. Further, research into AFNs for the early detection of HCC underscores their importance in elevating diagnostic precision. Employing aptamer-functionalized quantum dots as fluorescent markers allows for the specific identification of HCC cells [58], yielding highly sensitive and specific tumor detection. Such advancements present a promising new avenue for early HCC detection, potentially improving patient prognosis and therapeutic outcomes.

While AFNs showcase remarkable potential in HCC treatment, their development is met with several challenges. A key concern in ongoing research is the biocompatibility and potential toxicity associated with AFNs. Though nanomaterials may inherently be biocompatible, the addition of aptamers and functional molecules raises safety considerations, particularly regarding immunogenicity and toxicity. It's imperative to conduct in-depth investigations into the in vivo stability and biodistribution of AFNs. Additionally, the promise of AFNs to enhance therapeutic outcomes hinges on their ability for precise targeting of HCC cells, minimizing off-target effects and nonspecific accumulation. Technologically, synthesizing AFNs and scaling production pose significant hurdles due to the complexity of chemical processes involved, which challenge reproducibility and large-scale manufacturing. Ensuring batch-to-batch consistency is crucial for clinical applications, pressing the need for innovative, cost-effective, and scalable production methods tailored for AFNs. Addressing these challenges is vital for advancing AFNs from the laboratory to clinical settings, highlighting the necessity for interdisciplinary efforts in research and development.

Addressing the identified challenges necessitates comprehensive research across multiple domains. It's imperative to prioritize research on the biocompatibility and toxicity of AFNs, ensuring their safety and efficacy through rigorous preclinical and clinical evaluations. Enhancing the specificity and delivery mechanisms of AFNs through improved design and synthesis methods is critical for boosting their therapeutic efficiency. Moreover, the development of scalable and cost-effective synthesis strategies and production technologies is essential to meet clinical application demands and reduce AFNs' production costs. Encouraging interdisciplinary collaborations is crucial for harnessing diverse expertise from materials science, biology, and medicine. Such collaborative efforts are instrumental in fostering the innovative development and application of AFNs in treating hepatocellular carcinoma, highlighting the importance of a multidisciplinary approach in advancing this promising therapeutic technology.

In summary, AFNs present novel perspectives and opportunities in the treatment of HCC. Addressing the existing challenges and limitations is pivotal for the broad implementation of AFNs in HCC therapy, offering patients treatment modalities that are not only more targeted and efficient but also characterized by a reduced risk of adverse effects. The continuous progression of technology coupled with in-depth research endeavors is anticipated to unlock the full potential of AFNs within the domain of liver cancer therapy. This advancement promises to enrich the arsenal of therapeutic options available for HCC patients significantly, underscoring the critical role of innovative approaches and interdisciplinary collaboration in the field of oncology.

## Data Availability

Not applicable.

## Abbreviations

HCC: Hepatocellular carcinoma
AFNs: Aptamer-functionalized nanomaterials
CT: Computed tomography
MRI: Magnetic resonance imaging
SELEX: Systematic evolution of ligands by exponential enrichment
EPR: Enhanced permeability and retention
AFP: Alpha-fetoprotein
ELISA: Enzyme-linked immunosorbent assay
PBA: Phenylboronic acid
BMH: Bio-inspired MnO2 mixed hydrogel
TME: Tumor microenvironment
MNPs: Magnetic nanoparticles
QDs: Quantum dots
CNTs: Carbon nanotubes
SiNPs: Silicon dioxide nanoparticles
MSNs: Mesoporous Silica nanoparticles
CQDs: Carbon quantum dots
US: Ultrasound
PET: Positron emission computed tomography
SPECT: Single photon emission computed tomography
FI: Fluorescence imaging
GP73: Golgi protein 73
FRET: Fluorescence resonance energy transfer
NGQDs: Nitrogen-doped graphene quantum dots
NCL: Nucleolin protein
CDs: Carbon dots
AuNCs: Golden nanoclusters
CS: Chitosan
MTX: Methotrexate
GQDs: Graphene quantum dots
SPIONs: Superparamagnetic iron oxide nanoparticles
USPIOs: Ultrasmall superparamagnetic iron oxide nanoparticles
ICG: Indocyanine green
NLCs: Nanoliposomes
PTX: Paclitaxel
*S.*: Subcutaneous xenotransplantation model
*O.*: Orthotopic transplantation model
BPQDs: Black phosphorus quantum dots
BMSF: BPQD-hybridized mesoporous silica framework
TACE: Transcatheter arterial chemoembolization
SRF: Sorafenib
FDA: Food and drug administration
SD: Sprague dawley
NK: Natural killer
PLGA: Poly(lactic-co-glycolic acid
TPGS: D-α-Tocopherol polyethyleneglycol succinate
HMSNs: Hollow mesoporous silica nanoparticles
VRM: Viramidine
PEG: Polyethylene glycol
CD: Cyclodextrin
UA: Ursolic acid
EGCG: Epigallocatechin gallate
TILs: Tumor infiltrating lymphocytes
SiRNA: Small interfering RNA
ABC: ATP-binding cassette
MiRNAs: MicroRNAs
PDT: Photodynamic therapy
PTT: Photothermal therapy
DDS: Drug delivery system
ROS: Reactive oxygen species
UV: Ultraviolet
NIR: Near-infrared
SLNs: Solid lipid nanoparticles
OXA: Oxaliplatin
SAL: Salinomycin
5-FU: 5-Fluorouracil
miR-122: MicroRNA-122
Trem2: Trigger receptor-2
ICT: Icartin
USI: Sorafenib, ursolic acid, and indocyanine green condensed nanodrug
USFe^3+^ LA NPs: Ferroptosis-mediated metal-coordinated carrier-free nanodrug
FITC: Fluoresceine isothiocyanate
